# Characterization of a Pyranose Oxidase/*C*-Glycoside Oxidase from *Microbacterium* sp. 3H14, Belonging to the Unexplored Clade II of Actinobacterial POx/CGOx

**DOI:** 10.3390/biom14121510

**Published:** 2024-11-26

**Authors:** Andrea Martschini, Anja Kostelac, Dietmar Haltrich, Clemens K. Peterbauer

**Affiliations:** 1 Food Biotechnology Laboratory, Department of Food Science and Technology, BOKU University, 1190 Vienna, Austria; andrea.martschini@students.boku.ac.at (A.M.); anja.kostelac@boku.ac.at (A.K.); clemens.peterbauer@boku.ac.at (C.K.P.); 2 Doctoral Programme Molecular Biotechnology of Proteins BioToP, BOKU University, 1190 Vienna, Austria

**Keywords:** pyranose oxidase, FAD-dependent *C*-glycoside oxidase, AA3, kinetics

## Abstract

Pyranose oxidase (POx) is an FAD-dependent oxidoreductase and belongs to the glucose–methanol–choline (GMC) superfamily of oxidoreductases. As recently reported, POxs and FAD-dependent *C*-glycoside oxidases (CGOxs) share the same sequence space, and phylogenetic analysis of actinobacterial sequences belonging to this shared sequence space showed that it can be divided into four clades. Here, we report the biochemical characterization of a POx/CGOx from *Microbacterium* sp. 3H14 (*M*POx), belonging to the hitherto unexplored clade II of actinobacterial POx/CGOx. Overall, *M*POx demonstrates comparable features to POxs/CGOxs of clades III and IV, including the preference for glycosides over monosaccharides as electron donors. However, as *M*POx efficiently oxidizes the *C*-glycoside aspalathin as well as the *O*-glycoside phlorizin, it shows activity with yet another set of glycoside structures compared to other POx/CGOx members.

## 1. Introduction

The Carbohydrate-Active enZYme (CAZy) database (http://www.cazy.org/) comprises enzymes and associated modules (carbohydrate-binding modules) that are important for the breakage, modification or formation of glycosidic bonds, as well as auxiliary activity (AA) enzymes. AAs are redox enzymes assisting other CAZymes. The AA3 family consists of flavin adenine dinucleotide (FAD)-dependent enzymes belonging to the glucose–methanol–choline (GMC) oxidoreductase superfamily. It can be further divided into four subfamilies: AA3_1 (mostly cellobiose dehydrogenases), AA3_2 (including aryl alcohol oxidases and glucose oxidoreductases), AA3_3 (alcohol oxidases), and AA3_4 (pyranose oxidases) [1,2]. Enzymes of the AA3 family play a role in lignocellulose deconstruction. While they do not directly depolymerize lignocellulose, they provide H_2_O_2_ and/or hydroquinones for further enzymatic reactions [3].

Pyranose oxidases (POxs; EC 1.1.3.10) preferentially oxidize d-glucose and to a lower extent various other monosaccharides, including d-xylose, d-galactose, and l-sorbose [4,5]. Oxidation of substrates occurs primarily at the C2 position of these sugars to form the corresponding 2-ketoaldoses and the reduced cofactor FADH_2_. Subsequently, FADH_2_ is re-oxidized to FAD under simultaneous reduction of molecular oxygen to hydrogen peroxide [6]. Although POxs show pronounced oxidase activity, an additional distinct dehydrogenase activity is commonly observed as well. Alternative electron acceptors include 2,6-dichlorophenolindophenol (DCIP), 1,4-benzoquinone (BQ), and the ferrocenium ion (Fc). In some instances, the dehydrogenase activity is in fact stronger than the oxidase activity, as judged by the catalytic efficiencies [7,8].

Pyranose oxidases are found both in wood-degrading fungi and in various bacteria, while other members of the AA3 family are restricted to the kingdom of fungi [3,7]. POx was first mentioned in 1968 as a carbohydrate oxidase discovered in *Polyporus obtusus* [9]. Since then, various POxs of fungal origin have been reported and biochemically as well as structurally characterized, including POx from *Trametes multicolor* (*Tm*POx) and *Phanerochaete chrysosporium* (*Pc*POx) [4,5,10,11]. Although fungal POxs have been investigated for many years, characterization of bacterial POxs was reported only recently [8,12,13].

In 2021, it was shown that an FAD-dependent *C*-glycoside 3-oxidase (CGOx; EC 1.1.3.50) from *Microbacterium* sp. 5-2b (CarA) is closely related to bacterial POxs. CarA and homologous enzymes from *Arthrobacter globiformis* (*Ag*CarA) and *Microbacterium trichothecenolyticum* (*Mt*CarA) oxidize the sugar moiety of various *C*-glycosides and to a lower extent of *O*-glycosides at the C3 position to the corresponding 3-keto *C/O*-glycosides [14]. Kumano et al. [13] reported that the sequence of *Mt*CarA can indeed be found within a POx phylogenetic tree, and that POxs and CGOxs share the same sequence space. In this regard, it was shown that bacterial POxs not only oxidize various *C*- and *O*-glycosides but also have a preference for glycosides over monosaccharides [13].

Recently, we reported a detailed phylogenetic analysis of sequences belonging to the POx/CGOx shared sequence space, limiting the analysis to sequences of actinobacterial (Actinomycetota) origin since previously characterized bacterial enzymes of this sequence space are exclusively from species of this phylum [15]. This analysis showed that the phylogeny of POx/CGOx can be separated into four clades. So far, POxs/CGOxs of this sequence space have been biochemically characterized from *Kitasatospora aureofaciens* (*Ka*POx, clade I), *Streptomyces canus* (*Sc*POx, clade III), *M. trichothecenolyticum* (*Mt*CarA, clade IV), and *Pseudarthrobacter siccitolerans* (*Ps*G3Ox, clade IV) [8,13,14,16]. Interestingly, the homodimeric *Ka*POx was found to be more closely related to the homotetrameric fungal POxs than to the other characterized bacterial POxs/CGOxs. Moreover, *Ka*POx covalently binds FAD and does not show activity with glycosides like their fungal counterparts [8,15]. In contrast, *Sc*POx, *Ps*G3Ox, and *Mt*CarA are monomeric enzymes with a non-covalently bound FAD and a substrate preference for various glycosides and only low or negligible activity with monosaccharides [13,14,16]. In addition to the present-day members, several resurrected actinobacterial ancestors were characterized to study the evolution of substrate preferences among the different clades [15]. However, no extant POx has been characterized from clade II so far.

Here, we report the biochemical characterization of a POx/CGOx from *Microbacterium* sp. 3H14 (*M*POx) belonging to this yet unexplored clade II of the POx/CGOx sequence space. Characterization of *M*POx included determination of its oligomeric state, identification of the substrate scope, analysis of apparent steady-state kinetic parameters of selected substrates, stability measurements, and structural modelling and bioinformatical analysis. In this regard, our study provides further insights into the common features and different properties found among actinobacterial POxs/CGOxs.

## 2. Materials and Methods

### 2.1. Chemicals, Media, and Buffers

Bacterial cultures were grown in Luria–Bertani (LB) broth (10 g L^−1^ peptone from casein, 10 g L^−1^ NaCl, 5 g L^−1^ yeast extract) supplemented with 100 µg mL^−1^ ampicillin. For cell lysis, purification and storage of protein the following buffers were used: lysis buffer, 50 mM Tris-HCl, 10% glycerol, 0.1% Triton X-100, 2 mM MgCl_2_, 10 mM imidazole, 100 mM NaCl, 100 µg mL^−1^ lysozyme from chicken egg white (Sigma-Aldrich, St. Louis, MO, USA) and 1 U mL^−1^ DNase I (Sigma-Aldrich); buffer A, 100 mM NaCl, 30 mM imidazole, 5% glycerol, 50 mM Tris-HCl pH 7.5; buffer B, 100 mM NaCl, 500 mM imidazole, 5% glycerol, 50 mM Tris HCl pH 7.5; storage buffer, 100 mM NaCl, 10% glycerol, 50 mM Tris-HCl pH 7.5; and SEC buffer, 150 mM NaCl, 50 mM Tris-HCl pH 7.5. Britton–Robinson buffer (2.47 g L^−1^ boric acid, 2.7 mL L^−1^ phosphoric acid (85%), and 2.3 mL L^−1^ acetic acid (99%)) was used as a universal buffer for pH optimum and pH stability measurements. All chemicals were from Carl Roth (Karlsruhe, Germany), unless stated otherwise.

### 2.2. Gene Expression, Cultivation, and Cell Lysis

Recombinant *M*POx was produced in *E. coli* T7 Express (New England Biolabs, Ipswich, MA, USA). Chemically competent cells were transformed with a pET-21a(+) expression vector containing the coding sequence of *M*POx (NCBI Reference Sequence: WP_134352214.1) cloned between the restriction sites *Nde*I and *Hind*III. The construct further contained the sequence for a C-terminal His_6_ tag and the ampicillin resistance gene. Cells were cultured at 37 °C and 130 rpm agitation in 500 mL LB medium and inoculated with 10 mL of an overnight culture. When an OD_600_ of 0.6–1.0 was reached, gene expression was induced by adding isopropyl β-D-1-thiogalactopyranoside (IPTG) to a final concentration of 500 µM. The incubation temperature was reduced to 18 °C and expression lasted for approximately 18–20 h. Cultures were centrifuged (5000 rpm, 8 °C, 20 min, centrifuge Beckman Coulter Avanti J-26 XP, Brea, CA, USA, rotor JA-10), and the cell pellet was collected. For cell lysis, pellets from a total of 5 L medium were resuspended in lysis buffer to a final volume of 45 mL. Cells were kept on ice and lysed by sonication (sonicator Sonoplus HD60, Bandelin, Berlin, Germany) at 80 V and a 30% cycle for 6 times 5 min with 5 min breaks. Finally, the cell lysate was centrifuged (20,000 rpm, 4 °C, 100 min, centrifuge Beckman Coulter Avanti J-26 XP, rotor JA 25.50), the supernatant was collected, and 100 µL of a flavin adenine dinucleotide (FAD; 100 mM) solution were added for reconstitution of the enzyme.

### 2.3. Immobilized Metal Affinity Chromatography

Recombinant His-tagged enzymes were purified by immobilized metal ion affinity chromatography (IMAC). A pre-packed 5 mL HisTrap^TM^ column (Cytiva, Marlborough, MA, USA) was equilibrated with buffer A. The supernatant of cell lysis was loaded at a flow rate of 2 mL/min^−1^ and the column was washed with buffer A. For elution, a linear gradient from 0% to 100% buffer B within 10 min was set at a flow rate of 1 mL/min^−1^, and fractions of interest were pooled. Protein was concentrated and the buffer exchanged to storage buffer using Amicon tubes (molecular weight cut-off (MWCO) 30 kDa; Amicon ultra-15 centrifugal filter units, Millipore, Burlington, MA, USA) at 4000 rpm and 4 °C (centrifuge 5810 R, Eppendorf, Hamburg, Germany).

### 2.4. Size Exclusion Chromatography

For further purification and determination of the oligomeric state, protein preparations after IMAC were subjected to size exclusion chromatography (SEC) using a 120 mL Superdex 75 column (Cytiva) with a flow rate of 1 mL/min^−1^. Fractions of interest belonging to the same peak were pooled, filled up with storage buffer in an Amicon tube (MWCO 30 kDa), and concentrated by centrifugation (4000 rpm, 4 °C; centrifuge 5810 R, Eppendorf). In order to calculate the molecular weight of eluted protein, a calibration curve was constructed. The void volume (V_0_) was determined with 2 mg/mL^−1^ blue dextran (2000 kDa; dissolved in deionized water containing 5% glycerol). As protein standards, POx from *Trametes multicolor* (*Tm*POx, 270 kDa), β-amylase (200 kDa), albumin (66 kDa), POx from *Deinococcus aerius* (*Da*POx, 55 kDa), HRP (40 kDa), and carbonic anhydrase (29 kDa) were used. *Tm*POx and *Da*POx were previously produced in-house. Blue dextran, β-amylase, albumin, and carbonic anhydrase were taken from the kit for Molecular Weights 29,000–700,000 Da (Sigma-Aldrich), and the calibration curve was calculated according to the manufacturer’s instructions. Purified protein was stored at −80 °C in storage buffer. The theoretical molecular weight and the extinction coefficient of recombinant *M*POx were calculated using the Expasy ProtParam tool (https://web.expasy.org/protparam/; last accessed on 29 February 2024) [17].

### 2.5. SDS-PAGE

For SDS-PAGE, samples were mixed with 2 × Laemmli buffer (Bio-Rad, Hercules, CA, USA) and incubated for 5 min at 100 °C (AccuBlock^TM^ Digital Dry Bath, Labnet, Cary, NC, USA). The electrophoresis system, the precast gels (Mini-PROTEAN TGX Stain-Free Precast gels (4–16%)), and the marker (Precision Plus Protein^TM^ unstained protein standard) were from Bio-Rad. Generally, a voltage of 80 V was applied for 10 min, followed by 180 V for 50 min. For visualization, the ChemiDoc Imaging System XRS+ (Bio-Rad) was used.

### 2.6. Mass Spectrometry

The SDS-PAGE gel with samples from SEC was stained with Coomassie blue, and bands were selected for analysis by mass spectrometry. Mass spectrometry was performed by the Core Facility Mass Spectrometry (Rudolf Figl and Dr. Clemens Grünwald-Gruber; BOKU, Vienna, Austria). Peptide analysis was conducted in the following way: The samples were digested in-gel. Proteins were S-alkylated with iodoacetamide and digested with trypsin (Promega, Madison, WI, USA). The digested samples were loaded onto a nanoEase C18 column (nanoEase M/Z HSS T3 Column, 100 Å, 1.8 µm, 300 µm × 150 mm, Waters, Milford, MA, USA) using 0.1% formic acid as the aqueous solvent. A gradient from 1% B (B: 80% acetonitrile, 0.1% formic acid) to 40% B in 50 min was applied, followed by a 10 min gradient from 40% B to 95% B that facilitates elution of large peptides, at a flow rate of 6 µL/min^−1^. Detection was performed with an Orbitrap MS (Exploris 480, Thermo Fisher Scientific, Waltham, MA, USA) equipped with the standard H-ESI source in positive ion, DDA mode (=switching to MSMS mode for eluting peaks). MS scans were recorded (range: 350–1200 Da) and the 20 highest peaks were selected for fragmentation. Instrument calibration was performed using Pierce FlexMix Calibration Solution (Thermo Scientific). The analysis files were analyzed using PEAKS, which is suitable for performing MS/MS ion searches. The files were searched against an *E. coli* database.

### 2.7. Spectroscopic Analysis

Protein spectra of *M*POx were recorded with a diode array spectrophotometer (Agilent, Santa Clara, CA, USA) in quartz cuvettes (3 mm path length) in the range of 190.0–1100.0 nm. The absorbance at 280 nm (A_280_) was used to determine the protein concentration (*ε*_280,protein_ = 48,025 M^−1^ cm^−1^). As FAD also absorbs light at 280 nm, the extinction coefficient of the apoprotein was corrected by addition of the extinction coefficient of FAD (*ε*_280,FAD_ = 22,000 M^−1^ cm^−1^). FAD occupancy was calculated from the ratio of the concentration of FAD-loaded protein (*ε*_450, FAD_ = 11,300 M^−1^ cm^−1^) to total protein. For the determination of covalent FAD binding, spectra of the protein sample solution were measured with and without precipitation with HCl. To this end, 60 µL protein solution were mixed with 3 µL 10% HCl, incubated at 20 °C for 30 min (ThermoMixer F1.5, Eppendorf), and centrifuged for 5 min at 15,000 rpm (centrifuge 5424 R, Eppendorf). Spectra of supernatant after precipitation and 60 µL protein of the same concentration mixed with 3 µL storage buffer were measured. The ratio of the A_450_-value after HCl precipitation to the value without precipitation was calculated.

### 2.8. Activity Assays and Substrate Screening

For measurements of the oxidase activity, the AmplexRed (10-acetyl-3,7-dihydroxyphenoxazine; abcr, Karlsruhe, Germany)-coupled assay was used as the standard assay, according to the following procedure unless stated otherwise. Assays were performed at 200 µL scale in 96-well plates with a path length of 0.65 cm. Reaction mixtures consisted of 0.05 mM AmplexRed (ε_560_ = 54 mM^−1^ cm^−1^), 7.15 U mL^−1^ horseradish peroxidase (HRP; Sigma-Aldrich), variable amounts of substrate (electron donor), buffer (50 mM Tris-HCl pH 7.5), and enzyme. Reaction mixtures were shaken for 5 s before the change in absorbance was followed at 560 nm and 30 °C with an EnSpire Multimode plate reader (Perkin Elmer, Waltham, MA, USA). All samples and blanks were measured in triplicate. Dehydrogenase activity assays were conducted following the same procedure as the AmplexRed assay but replacing AmplexRed and HRP with 0.15 mM of 2,6-dichlorophenolindophenol (DCIP, measured at 520 nm, ε_520_ = 6.9 mM^−1^ cm^−1^; Fluka), 0.5 mM of 1,4-benzoquinone (BQ, measured at 290 nm, ε_290_ = 2.24 mM^−1^ cm^−1^; Sigma-Aldrich), or 1 mM of ferrocenium hexafluorophosphate (Fc, measured at 300 nm, ε_300_ = 4.3 mM^−1^ cm^−1^; Santa Cruz Biotechnology, Dallas, TX, USA) as electron acceptors. The monosaccharides d-glucose and d-xylose (Fluka, Buchs, Switzerland); the *C*-glycosides aspalathin, homoorientin (TargetMol, Boston, MA, USA), isovitexin (TargetMol), puerarin (abcr), mangiferin (Sigma-Aldrich), and carminic acid (Glentham Life Science, Glentham, UK); the *O*-glycosides phlorizin (abcr), fraxin (BLDpharm, Shanghai, China), rutin (abcr), and salicin (Thermo Fisher Scientific); and the *S*-glycoside sinigrin (Carl Roth) were tested as potential electron donor substrates. Monosaccharide substrate stocks were prepared in deionized water, while glycoside substrates stocks were dissolved in DMSO (Thermo Fisher Scientific). Initial screening for oxidase and dehydrogenase activity of *M*POx was performed using the AmplexRed and DCIP assays with d-glucose (at concentrations of 20 mM and 200 mM) and aspalathin, fraxin, and homoorientin (at concentrations of 0.02 mM and 0.2 mM each) as substrates, with final protein concentrations of 0.01 mg mL^−1^ and 0.001 mg mL^−1^. For further screening, final substrate concentrations of 200 mM for monosaccharides and 0.2 mM for glycosides with a final protein concentration of 0.1 mg mL^−1^ were selected.

### 2.9. Kinetic Analysis

Apparent steady-state kinetic parameters for the monosaccharides d-glucose and d-xylose and the glycosides aspalathin and phlorizin as electron donors were determined, using molecular oxygen as an electron acceptor at a constant concentration (ambient air). The standard setup of the AmplexRed assay was used with varying substrate concentrations (d-glucose 0.02–50 mM, d-xylose 5–400 mM, aspalathin and phlorizin 0.002–3 mM), and a final protein concentration of 0.1 mg mL^−1^. The Michaelis constant (*K*_m_), the maximum velocity (*v*_max_), and, in case of substrate inhibition, the inhibition constant (*K*_i_) were determined by plotting the specific activity (*v*) versus the substrate concentration ([S]). Curves for d-glucose and d-xylose were fitted with SigmaPlot 15.0 (Systat Software, Inc., Frankfurt am Main, Germany) to the Michaelis–Menten Equation (1):

(1) $$v=\frac{v_{max}\;\times \;[S]}{K_{m}+[S]}$$

For aspalathin and phlorizin, substrate inhibition curves (Equation (2)) were calculated with OriginPro (version 2024, OriginLab Corporation, Northampton, MA, USA).

(2) $$v=\frac{v_{max}\;\times \;[S]}{K_{m}+\left[S\right]\;\times \;\frac{1+[S]}{K_{i}}}$$

Finally, catalytic constants (*k*_cat_) as well as the catalytic efficiencies (*k*_cat_/*K*_m_) were calculated for all substrates.

### 2.10. Stability Measurements

The thermal transition temperature (melting temperature *T*_m_) of *M*POx was determined using the *Thermo*FAD assay [18]. Measurements were performed by measuring cofactor fluorescence in a real-time PCR cycler (iCycler, Bio-Rad) in the range of 20 °C to 75 °C with increments of 1 °C per minute. Samples of purified protein (25 µL of 1 mg mL^−1^) were measured in triplicate. All other stability measurements (pH stability, thermostability (*T*_50_^30’^), and half-life times (*t*_1/2_ at 40 °C and 50 °C)) were conducted using the standard setup of the AmplexRed assay, with final concentrations of 0.2 mM phlorizin as the substrate and protein concentrations of 0.05 mg mL^−1^ and 0.025 mg mL^−1^. For determination of the pH optimum and pH stability, common buffers as well as universal buffer in a pH range of 5.0–9.0 with increments of 0.5 units were used. Common buffers (50 mM each) were acetate buffer (pH 5.0–6.0), potassium phosphate buffer (pH 6.0–7.0), and Tris-HCl (pH 7.0–9.0). For pH stability, the protein was incubated (ThermoMixer F1.5, Eppendorf) in various buffers and pH values for 30 min at 30 °C and left on ice for 10 min, and then the residual activity was measured at pH 7.5 (50 mM Tris-HCl) under standard assay conditions. Half-life times were determined by incubating the enzyme in a ThermoMixer F1.5 (Eppendorf) at 40 °C (0–200 min) and at 50 °C (0–100 min), taking samples at various time points. Samples were left on ice for 10 min, and residual activity was measured. Data were fitted to a linear regression curve according to the following Equation (3):

(3) $$\ln\left(relative\;activity\right)={ln(relative\;activity)}_{0}-k_{d}\;\times \;t$$

Determination of the rate constant (*k*_d_) further allowed the calculation of half-life times with Equation (4).

(4) $$t_{1/2}=\frac{ln(2)}{k_{d}}$$

Samples for the determination of the *T*_50_^30’^-value were incubated for 30 min in a thermal cycler (C1000 Touch Thermal Cycler, Bio-Rad) using temperature gradients in the range of 30–54 °C. For incubation at 20 °C and 25 °C a ThermoMixer F1.5 (Eppendorf) was used, and another sample was incubated at 4 °C. Samples were cooled down on ice for 10 min and the residual activity was determined by the standard assay. Data were fitted to a 3-parameter sigmoidal regression curve (Equation (5)) with the parameters a, b, and x_0_. The *T*_50_^30’^-value was determined as the temperature where the residual activity equals 50%.

(5) $$residual\;activity=\frac{a}{1+e^{\frac{-T-x_{0}}{b}}}$$

Graphical representation of data was performed with SigmaPlot (SigmaPlot v.15.0, Systat Software Inc.).

### 2.11. Structural Modelling and Bioinformatic Analysis

A structural model of *M*POx was calculated with RoseTTAFold (https://robetta.bakerlab.org, accessed on 28 November 2023) and compared to known structures of related enzymes, accessed via the RCSB protein data bank (PDB, https://www.rcsb.org, accessed on 3 December 2023). For comparison, structures of fungal POxs from *Phanerochaete chrysosporium* (*Pc*POx; PDB accession number 4MIF [11]) and *Trametes multicolor* (*Tm*POx; PDB 1TT0 [10]), as well as bacterial POxs/CGOxs from *Microbacterium trichothecenolyticum* (*Mt*CarA; PDB 7DVE [14]) and *Pseudarthrobacter siccitolerans* (*Ps*G3Ox; PDB 7QF8 [16]), were taken. Analysis of the structural model was carried out with PyMOL (PyMOL Molecular Graphics System version 2.4, Schrödinger, LLC, New York, NY, USA). In addition, SWISS-MODEL (https://swissmodel.expasy.org, accessed on 21 October 2024) was used to search for the most structurally similar proteins. Furthermore, a search for sequences similar to *M*POx (NCBI Reference Sequence: WP_134352214.1) was conducted using the Protein Basic Local Alignment Search Tool (blastp; https://blast.ncbi.nlm.nih.gov/; accessed on 12 December 2023) restricted to *Microbacterium* sp. 3H14, followed by a search for templates (https://swissmodel.expasy.org/; accessed on 12 December 2023) [19] for another GMC-oxidoreductase (*M*POx2; NCBI Reference Sequence: WP_134352227.1) found in the genome. Multiple sequence alignment with blastp (accessed on 17 April 2023) was used for pairwise alignment of *M*POx and *M*POx2 with other POx/CGOx sequences to determine the sequence identities of *M*POx and *M*POx2 with POx from *Streptomyces canus* (*Sc*POx; NCBI Reference Sequence: WP_062047964.1), *Ka*POx (UniProt accession A0A1E7NAU4), *Mt*CarA (UniProt accession A0A0M2HFA3), and *Ps*G3Ox (UniProt accession A0A024H8G7). Sequences of *M*POx, *Pc*POx, *Tm*POx, *Mt*CarA, *Ps*G3Ox, and *M*POx2 were aligned via multiple sequence alignment (MAFFT, https://ngphylogeny.fr/tools/, accessed on 18 April 2024) [20], and aligned sequences were compared in MEGA version 11 [21]. Signal peptide prediction was performed using TatP–1.0 and SignalP–6.0 (https://services.healthtech.dtu.dk; accessed on 12 March 2024 and 18 April 2024, respectively) [22,23]. Additionally, a search for open reading frames (ORFs) near the ORF of the target protein was carried out via the ORF finder (https://www.ncbi.nlm.nih.gov/orffinder/, accessed on 12 March 2024).

## 3. Results

### 3.1. Expression and Purification of Recombinant MPOx

The gene coding for *M*POx was heterologously expressed in *Escherichia coli* T7 Express, cells were lysed by sonication after the cultivation/induction, and the His-tagged protein was purified by immobilized metal ion affinity chromatography (IMAC). Presence of the protein after induction and during the purification together with the purity of purified protein (theoretical mass of 57 kDa) were confirmed by SDS-PAGE (Supplementary Figure S1). As we detected some impurities in the pooled fractions after IMAC, we performed an additional purification step, size exclusion chromatography (SEC). Surprisingly, the chromatogram of the SEC showed three major peaks. Screening for activity with phlorizin and oxygen as substrates revealed that only fractions of the second and third peak showed oxidase activity. Calculation of the molecular weight corresponding to these two peaks (Supplementary Figure S2) yielded 139 kDa for the second peak and 65 kDa for the third peak. These calculated molecular weights are well in the range of theoretical weights for *M*POx monomers and dimers (57 kDa and 114 kDa, respectively). Thus, *M*POx seems to form both monomers and dimers in solution. Peptide analysis by mass spectrometry showed that the target sequence of the *M*POx protein was in fact identified in both peaks 2 and 3, and that the impurity that was separated by SEC results from an *E. coli* host protein, the catabolite activator protein. Purified dimeric fractions were unstable after incubation for some time. When keeping the fraction after SEC containing only the dimers at 0.8 mg mL^−1^ for 5 days at 4 °C, we observed again both monomers and dimers after SEC. When incubating the monomer fraction after SEC under identical conditions, no dimers were seen after an SEC run, only monomers. Therefore, we assume that the dimeric state of *M*POx was only an artefact formed during the purification procedure. We continued all further experiments with the fraction corresponding to the monomeric form after SEC, to which we from this point on refer to as *M*POx. Spectrophotometric analysis of *M*POx (Supplementary Figure S3) showed the typical spectrum of a flavoprotein, with an absorption maximum at 280 nm for the polypeptide chain itself, and maxima around 390 nm and 450 nm, which are characteristic for FAD. FAD occupancy was calculated as the ratio of concentrations of FAD-loaded protein at A_450_ (*ε*_450, FAD_ = 11,300 M^−1^ cm^−1^) to overall protein at A_280_ (*ε*_280, protein_ = 48,025 M^−1^ cm^−1^ and *ε*_280, FAD_ = 22,000 M^−1^ cm^−1^). FAD occupancy was 90% for the purified protein, and this occupancy rate was taken into account for all further activity calculations.

### 3.2. Substrate Screening and Kinetic Analysis

**Figure 1:**
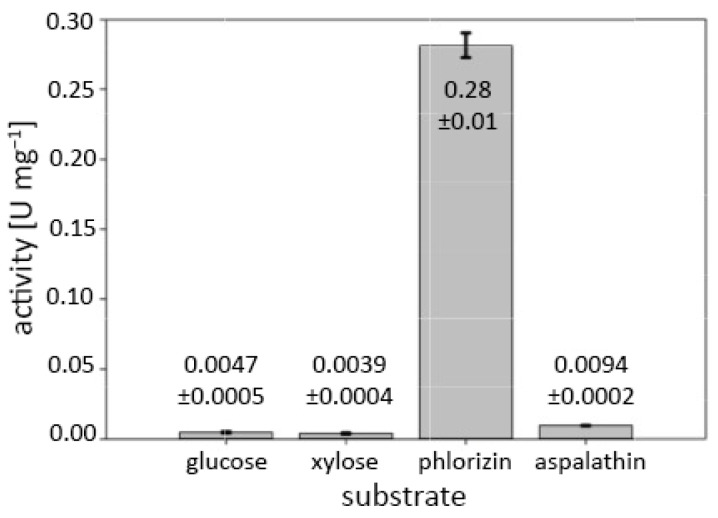
Initial screening for oxidase activities of *M*POx and different electron donor substrates. Final concentrations in the assay were 200 mM for monosaccharides (d-glucose, d-xylose) and 0.2 mM for glycosides (aspalathin, phlorizin). Reactions were performed at 30 °C and pH 7.5 (50 mM Tris-HCl).

Initial screening for oxidase and dehydrogenase activity of *M*POx was performed with molecular oxygen and 2,6-dichlorophenolindophenol (DCIP) as electron acceptors in combination with d-glucose, aspalathin, homoorientin, and fraxin as substrates (electron donors). Oxidase activity was observed with d-glucose and aspalathin, while no dehydrogenase activity with DCIP was detected. Further screening for dehydrogenase activity did not show any positive results with 1,4-benzoquinone (BQ) and the ferrocenium ion (Fc) as electron acceptors. Activity tests with monosaccharides (d-glucose, d-xylose), *C*-glycosides (aspalathin, homoorientin, isovitexin, puerarin, mangiferin, carminic acid), *O*-glycosides (fraxin, phlorizin, rutin, salicin), and one *S*-glycoside (sinigrin) were conducted in the presence of oxygen. These showed that *M*POx oxidizes aspalathin and phlorizin and less efficiently also d-glucose and d-xylose (Figure 1). Activity with all other substrates was negligible.

Apparent steady-state kinetic parameters were determined for d-glucose, d-xylose, aspalathin, and phlorizin with molecular oxygen (air) as the electron acceptor at a fixed concentration (Table 1). Data for monosaccharides were fitted to the Michaelis–Menten equation and data for glycosides to the substrate inhibition curve (Supplementary Figure S4). In general, *M*POx showed very low *K*_m_-values for glycosides, and substrate inhibition occurred at higher concentrations of the glycosides. For the monosaccharides, the *K*_m_-value for d-glucose was much lower than for d-xylose, resulting in a higher catalytic efficiency for d-glucose, although the catalytic constants for the monosaccharides were in the same range, even though they were very low.

### 3.3. Stability and Optima

**Figure 2:**
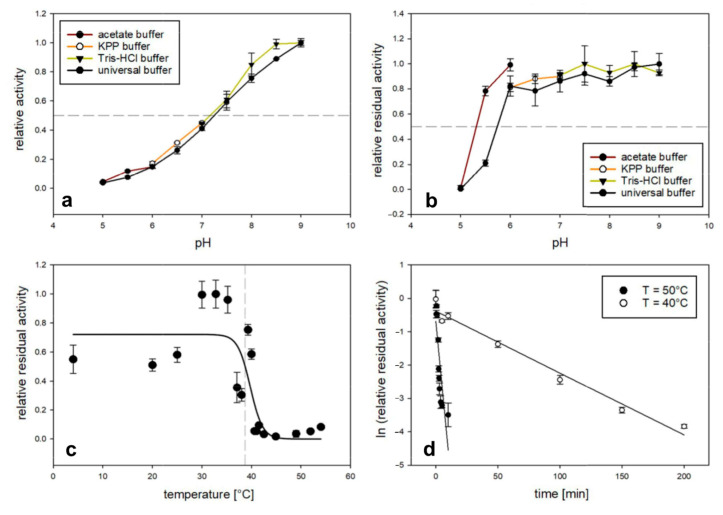
Activity optima and stability of *M*POx, measuring oxidase activity in triplicate with 0.2 mM phlorizin as substrate. (**a**) pH optimum at 30 °C. The dashed line indicates 50% relative activity. (**b**) pH stability at 30 °C. (**c**) Thermal stability (*T*_50_^30’^) at pH 7.5 (50 mM Tris-HCl). The dashed vertical line at 38.7 °C indicates 50% relative residual activity. (**d**) Half-life times (*t*_1/2_ at 40 °C and 50 °C) at pH 7.5 (50 mM Tris-HCl).

The thermal transition temperature (melting temperature, *T*_m_) of *M*POx was determined to be 42.2 °C (Supplementary Figure S5) using the *Thermo*FAD assay [18]. Measurements for the pH optimum showed that activity increased with the pH value and that at least 50% of the activity remained in the range of pH 7.5–9.0 (Figure 2a). pH stability was determined by incubation of an enzyme preparation for 30 min at 30 °C in different buffers of various pH and subsequent measurement of the residual activity under standard assay conditions. Under these conditions, more than 80% of the activity was retained when using common buffers at pH 5.5–9.0 and universal buffer at pH 6.0–9.0 (Figure 2b). At pH 5.0 and below, *M*POx precipitated regardless of the buffer. The *T*_50_^30’^-value was determined as another indicator of thermal stability (Figure 2c). The results show that ≥50% of the enzyme’s activity remained after incubation for 30 min at pH 7.5 (50 mM Tris-HCl) at temperatures ≤38.7 °C. Additionally, half-life times (*t*_1/2_) were assessed at 40 °C and 50 °C and equaled 37 min and 1.8 min, respectively (Figure 2d).

### 3.4. Sequence Analysis and Structural Modelling

**Figure 3:**
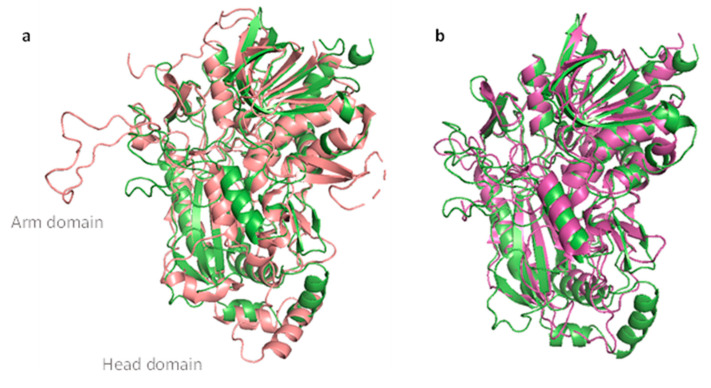
Structural model of *M*POx (green) aligned with the structures of (**a**) *Pc*POx (pink; monomeric subunit) and (**b**) *Mt*CarA (magenta).

Structural modelling of *M*POx was performed with RoseTTAFold, and the model was compared to crystal structures of fungal *Pc*POx (PDB accession number 4MIF) and *Tm*POx (PDB 1TT0), as well as of bacterial *Mt*CarA (PDB 7DVE) and *Ps*G3Ox (PDB 7QF8). RMSD values of these alignments were 1.385 Å for *Pc*POx, 1.403 Å for *Tm*POx, 1.367 Å for *Mt*CarA, and 1.377 Å for *Ps*POx. The overall structure was found to be more similar to the bacterial enzymes than to the fungal enzymes, as it lacked the typical head and arm domains of fungal POxs, as expected, since these latter features have been attributed to oligomerization of fungal POx (Figure 3). In addition, a search using SWISS-MODEL showed that MPOx is structurally most similar to the biochemically characterized CGOx *Mt*CarA. Kumano et al. [14] found that the α-helix of *Mt*CarA (residues 59–70) is shifted compared to the α-helix of a fungal Pox, as otherwise bulky substrate cannot bind since the aglycone part would clash with the helix. Comparison of the corresponding α-helix (residues 54–65) in the *M*POx structural model showed that the helix aligned well with the helix of *Mt*CarA and was also shifted compared to the helix in *Pc*POx.

**Figure 4:**
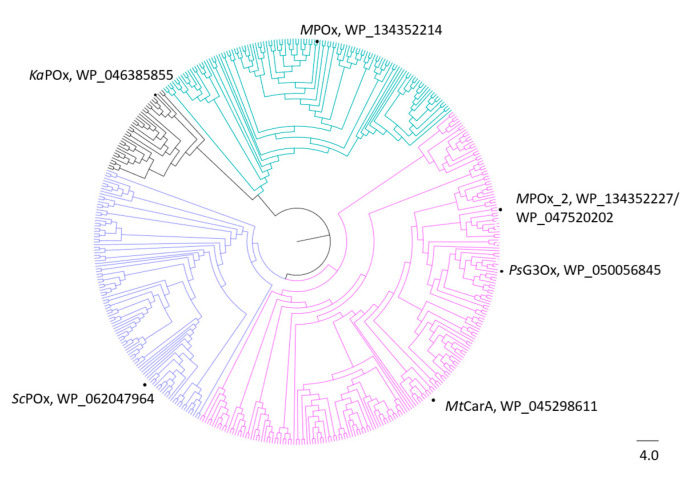
Phylogenetic tree representing the distribution of actinobacterial sequences. Clades were colored based on the origin of sequences: *Amycolatopsis* and *Streptomyces* in black (clade I), *Microbacterium* in cyan (clade II), *Streptomyces* in purple (clade III), and *Arthrobacter* and *Microbacterium* in pink (clade IV). Present-day enzymes that have been studied in more detail are indicated together with their accession numbers. The bar represents the phylogenetic distance as amino acid substitution per site.

The amino acid sequence of *M*POx was subjected to a blastp search restricted to *Microbacterium* sp. 3H14 to find homologous sequences within this organism. Another GMC oxidoreductase with a query cover of 97% and 40.19% identity was found. Subsequently, pairwise alignment of *M*POx and *M*POx2 with other characterized members of the POx/CGOx sequence space was performed (Supplementary Table S1). *M*POx shares 35–40% sequence identity with *Ps*G3Ox, *Mt*CarA, *Sc*POx, and *Ka*POx, which is in the same range as sequence identities of *M*POx2 with *Ka*POx and *Sc*POx. However, *M*POx2 had noticeably higher sequences identities to *Ps*G3Ox and *Mt*CarA (70–73%). This suggests that *M*POx2 is another POx/CGOx belonging to clade IV of the shared sequence space, and *M*POx2 was indeed positioned in the branch of clade IV in the phylogenetic tree (Figure 4).

Multiple sequence alignment (MAFFT) was performed for sequences of *M*POx, *Pc*POx, *Tm*POx, *Mt*CarA, *Ps*G3Ox, and *M*POx2 (Supplementary Figure S6). Comparison of amino acids aligning with the substrate loop of *Ps*G3Ox (^346^ASPVPLADD^354^) [16] showed that the amino acid sequence of *M*POx2 (^352^ASPIALAED^360^) was well comparable to sequences of *Mt*CarA (^350^ASPVKLADD^358^) and *Ps*G3Ox. In contrast, *M*POx (^362^NPPFQL^367^) had different amino acids and deletions in this loop, which reflects the different substrate preferences of these enzymes.

Lastly, as proteins such as *Ka*POx and *Tm*POx are known to be secretory proteins, TatP and SignalP were applied for signal peptide prediction for *M*POx, but the results indicate that *M*POx contained no signal peptide (Supplementary Figure S7). In addition, the genomic sequence in the region of the open reading frame (ORF) coding for *M*POx was analyzed using the ORF finder. Although several ORFs were identified, only one ORF was found upstream of the target protein, thus suggesting that no signal peptide or secretion is needed for the physiologically relevant role of *M*POx in nature.

## 4. Discussion

Although fungal pyranose oxidases have been studied for many years, bacterial POxs have been explored only recently [3] and have been found to share the same sequence space as CGOxs [13,14]. As recently shown by Kostelac et al. [15], the actinobacterial POx/CGOx sequence space can be divided into four clades. Previously characterized present-day members are found in clade I (*Ka*POx), clade III (*Sc*POx), and clade IV (*Ps*G3Ox, *Mt*CarA). Furthermore, several resurrected ancestors have been analyzed [15]. However, no extant member has been characterized in clade II so far. The objective of this study was to provide further insights into this shared sequence space by characterization of a POx from *Microbacterium* sp. 3H14 (*M*POx) belonging to this unexplored clade II.

In accordance with members of clade III (*Sc*POx) and clade IV (*Ps*G3Ox, *Mt*CarA) of the POx/CGOx actinobacterial sequence space [13,14,16], *M*POx is a monomeric protein with a non-covalently bound FAD-cofactor and a preference for glycosides over monosaccharides as its electron donor substrate. The only currently characterized member of clade I, *Ka*POx, shows distinct differences, as it is homodimeric with a covalently bound cofactor and has no activity with glycosides [8]. It should be noted that we also observed dimers for *M*POx, but as they were less stable and present in much lower amounts than monomers, we regard them as artefacts.

**Figure 5:**
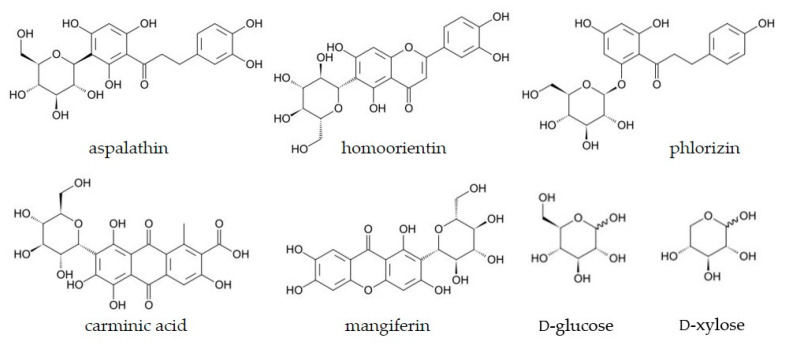
Structures of selected *C*- and *O*-glycosides and monosaccharides used as substrates by different actinobacterial POxs/CGOxs.

An initial substrate screening for electron donor and electron acceptor substrates revealed that *M*POx shows oxidase activity towards glycosides and to a significantly lower extent to monosaccharides, but no dehydrogenase activity (activity with DCIP, BQ, or Fc). Since Kostelac et al. [15] reported that dehydrogenase activity is negligible for the clade-II ancestor N67, it seems as if this activity were lost during evolution within that phylogenetic clade, although several other POxs are known to show both oxidase and dehydrogenase activity; in fact, their dehydrogenase activity can even be more pronounced than the reactivity with oxygen [8,13]. Out of eleven different glycosides tested, many of which were shown to be good substrates for other glycoside-oxidizing enzymes, *M*POx oxidized the glucose moiety of phlorizin and aspalathin. It reacted with the *O*-glycoside phlorizin at much higher rates than with the *C*-glycoside aspalathin, while *Sc*POx as well as CarA and its homologues showed higher catalytic efficiencies for *C*-glycosides [13,14]. Selected apparent steady-state kinetic constants for actinobacterial POx/CGOx members and for the closest resurrected ancestor N67 are summarized in Table 2. Structural formulas of selected substrates are provided in Figure 5. *M*POx shows kinetic parameters for aspalathin comparable to N67, while the ability to oxidize homoorientin seems to have been lost during the evolution of *M*POx, even though activity with homoorientin was reported for all resurrected ancestors as well as for the present-day enzymes *Sc*POx and *Ps*G3Ox. As characterized ancestors in clade IV and the extant member *Ps*G3Ox show high catalytic efficiencies for homoorientin [15], we assume that *M*POx2 can react with homoorientin. Furthermore, we observed considerable substrate inhibition of *M*POx with phlorizin and aspalathin, which was also reported for CarA and carminic acid (*K*_i_ = 0.07 ± 0.05 mM) [14].

*Microbacterium* sp. 3H14 was isolated from a plant-associated arctic rhizosphere in Norway (https://img.jgi.doe.gov/; accessed on 12 August 2024). The dihydrochalcone phlorizin (or phloridizin, as it is sometimes referred to) is a compound found in several plants, albeit in low concentrations. It is, however, the predominant phenolic compound in the apple tree, where it is found in the bark, roots, leaves, and fruits. Apple peel contains between 12 and 418 mg phlorizin per kg fresh material (corresponding to approx. 0.03–0.96 mM), and its concentration in the bark and leaves is much lower [24,25]. Phlorizin is thought to be involved in disease resistance of apple against various pathogens. Hence, *M*POx could have a defensive role for *Microbacterium* sp. 3H14 in removing a natural antimicrobial substance produced by plants, or by initiating the breakdown and utilization of phlorizin. It was recently shown that the first step in the biological degradation of both *C*- and *O*-glycosides is the oxidation of their sugar moieties by POx/CGOx, followed by a β-elimination reaction [26,27].

A structural model of *M*POx indicated that it shares the common structural features of POxs and that it is more similar to the monomeric bacterial POxs/CGOxs, such as *Mt*CarA (PDB 7DVE) and *Ps*G3Ox (PDB 7QF8), than to the single subunit of fungal POxs, e.g., *Pc*POx (PDB 4MIF) and *Tm*POx (PDB 1TT0). Furthermore, one specific α-helix near the active site entrance of *Mt*CarA is shifted compared to a fungal POx. Kumano et al. stated that this shift is necessary to accommodate glycosides, since its position in fungal POxs would clash with the aglycone of these bulky substrates [14]. As analyzed by multiple sequence alignment with MAFFT, amino acids of *M*POx that align with the proposed substrate loop of *Ps*G3Ox [16] are altered compared to those of *Ps*G3Ox and other clade IV members, supporting the idea that members of clade II of actinobacterial POx/CGOx cover a different substrate range than members of clade IV. In addition, we identified a second *pox/cgox* gene that belongs to clade IV in *Microbacterium* sp. 3H14, and it presumably has a different substrate scope than *M*POx. This indicates that bacteria can evolve several metabolic pathways for the degradation of glycosides, compounds that are found widely and in great structural diversity in nature.

## 5. Conclusions

In this paper, we report the first biochemical characterization of a pyranose oxidase/*C*-glycoside oxidase that belongs to the hitherto unexplored clade II of actinobacterial POx/CGOx, the enzyme from *Microbacterium* sp. 3H14 (*M*POx). Our studies show that *M*POx presents a substrate preference for a selected group of glycosides, while monosaccharides are poor substrates. This indicates that most actinobacterial POx/CGOx—the enzymes belonging to clades II to IV—show a substrate preference for glycosides, while only members of clade I show significantly different catalytic properties, as they oxidize monosaccharides efficiently, and negligible activity with glycosides. As only few members of bacterial pyranose oxidases/*C*-glycoside oxidases have been studied in detail, further studies could reveal biocatalysts with differing or novel properties.

## Figures and Tables

**Table 1:** Apparent steady-state kinetic parameters for different substrates of *M*POx. The concentration of the electron donor substrates varied, while the concentration of the electron acceptor oxygen held constant (air saturation). Activities were measured in triplicate at 30 °C and pH 7.5 (50 mM Tris-HCl).

**Substrate**	***K*** **_m_** **[mM]**	***K*** **_i_** **[mM]**	***k*** **_cat_** **[s^−1^]**	***k*** **_cat_** **/*K*_m_** **[s^−1^ M^−1^]**
**d**-glucose	0.66 ± 0.03	-	0.0047 ± 0.0001	7.10
**d**-xylose	19 ± 1	-	0.0038 ± 0.0001	0.20
Aspalathin	0.085 ± 0.014	0.30 ± 0.05	0.019 ± 0.002	220
Phlorizin	0.057 ± 0.006	0.48 ± 0.05	0.46 ± 0.03	8100

**Table 2:** Apparent steady-state kinetic parameters for various actinobacterial members of the POx/CGOx sequence space and their best identified glycoside substrate. All constants were determined as oxidase activity except for *Sc*POx, where DCIP was used as the electron acceptor. Measurements were generally performed at 30 °C and pH 7.5, except for *Ps*G3Ox (37 °C), CarA (pH 8.0), and for *Sc*POx in combination with d-glucose and d-xylose (pH 6.5).

Enzyme	Substrate	*K*_m_ [mM]	*k*_cat _ [s^−1^]	*k*_cat_/*K*_m_ [s^−1^ M^−1^]	Reference
***Ka*POx** (clade I)	d-glucose	1.5 ± 0.1	15 ± 0	1.0 × 10^4^	[8]
	d-xylose	32 ± 4	6.8 ± 0.3	210	
***M*POx** (clade II)	d-glucose	0.66 ± 0.03	0.0047 ± 0.0001	7.1	This study
	Phlorizin	0.057 ± 0.006	0.46 ± 0.03	8.1 × 10^3^	
**N67** (ancestor of clade II)	d-glucose	31 ± 8	0.020	0.65	[15]
	Homoorientin	0.12 ± 0.05	0.011	91	
	Asphalatin	0.052 ± 0.0007	0.027	520	
***Sc*POx** (clade III)	d-glucose	2100 ± 300	0.36	0.18	[13]
	d-xylose	1200 ± 200	0.09	0.07	
	Homoorientin	0.16 ± 0.04	0.065	410	[15]
**CarA** (clade IV)	Carminic acid	0.019 ± 0.001	4.3 ± 0.1	2.3 × 10^5^	[14]
***Ps*G3Ox** (clade IV)	d-glucose	460 ± 130	0.19 ± 0.03	0.45	[16]
	d-xylose	1000 ± 200	0.13 ± 0.02	0.13	
	Mangiferin	0.49 ± 0.10	8.13 ± 1.67	1.92 × 10^4^	

## Data Availability

The data that support the findings of this study are available from the corresponding author upon reasonable request.

## Supplementary Materials

The following supporting information can be downloaded at https://www.mdpi.com/article/10.3390/biom14121510/s1: Figure S1: SDS-PAGE to evaluate gene expression and protein purification by IMAC; Figure S2: Chromatogram of size exclusion chromatography of the protein sample after IMAC; Figure S3: UV-Vis spectrum of a purified MPOx sample; Figure S4: Michaelis–Menten curves for different substrates of MPOx; Figure S5: Determination of the melting temperature; Figure S6: Multiple sequence alignment; Figure S7: Signal peptide predictions; and Table S1: Sequence identities of MPOx and MPOx2 with other members of the actinobacterial sequence space.
