# Supplementary material for: Characterization of a Pyranose Oxidase/C-Glycoside Oxidase from Microbacterium sp. 3H14, Belonging to the Unexplored Clade II of Actinobacterial POx/CGOx
Source: Biomolecules. 2024 Nov 26;14(12):1510. doi: 10.3390/biom14121510 (PMC11673046; doi:10.3390/biom14121510)
Supplement: Supplementary file 1 [file biomolecules-14-01510-s001.zip › biomolecules-3232536-supplementary.pdf]

biomolecules

MDPI

Supplementary Material

Characterization of a Pyranose Oxidase/*C*-Glycoside Oxidase from *Microbacterium* sp. 3H14, Belonging to the Unexplored Clade II of Actinobacterial POx/CGOx

Andrea Martschini [1], Anja Kostelac [1,2], Dietmar Haltrich [1,*] and Clemens K. Peterbauer [1]

1 Food Biotechnology Laboratory, Department of Food Science and Technology, BOKU University, 1190 Vienna, Austria; andrea.martschini@students.boku.ac.at (A.M.); anja.kostelac@boku.ac.at (A.K.); clemens.peterbauer@boku.ac.at (C.K.P.)
2 Doctoral Programme Molecular Biotechnology of Proteins BioToP, BOKU University, 1190 Vienna, Austria
* Correspondence: dietmar.haltrich@boku.ac.at

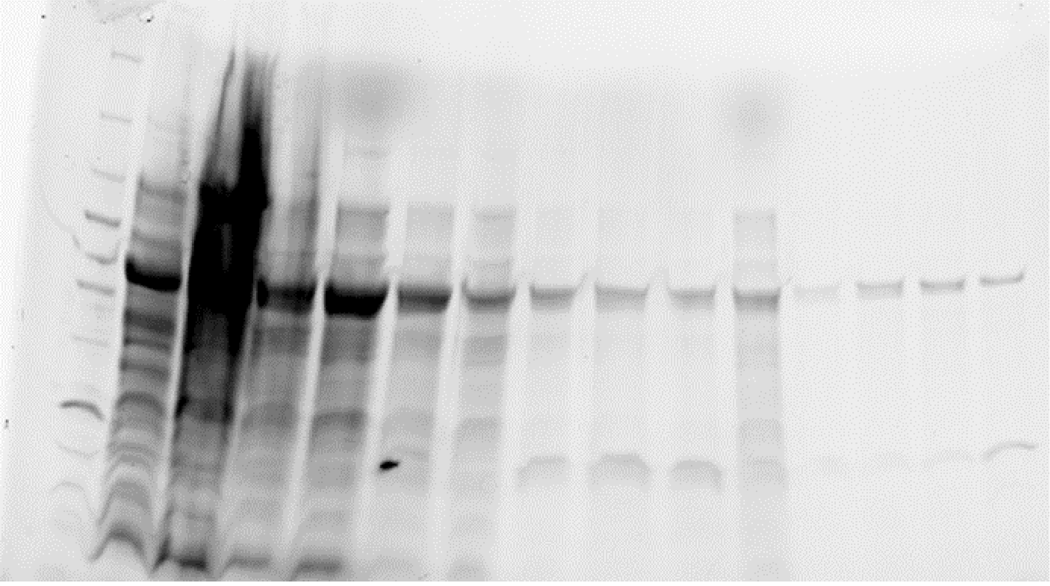

Figure S1. SDS-PAGE to evaluate gene expression and protein purification by IMAC. Assigned lanes: 1) marker; 2) whole cells; 3) supernatant after cell lysis; 4) cell pellet after cell lysis; 5) first flow-through; 6) F10; 7) F11; 8) F12; 9) F13; 10) F17; 11) second flow-through; 12) F30; 13) F31; 14) F32; 15) pooled fractions (F9-16 and 29-31).

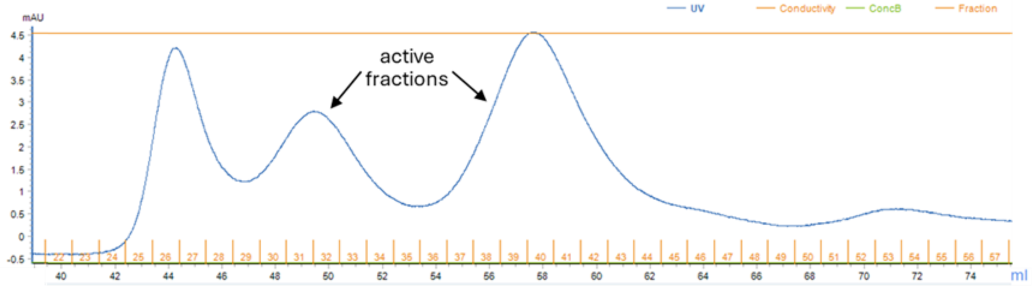

Figure S2. Chromatogram of size exclusion chromatography of the protein sample after IMAC. Only fractions belonging to the second and third peak showed activity.

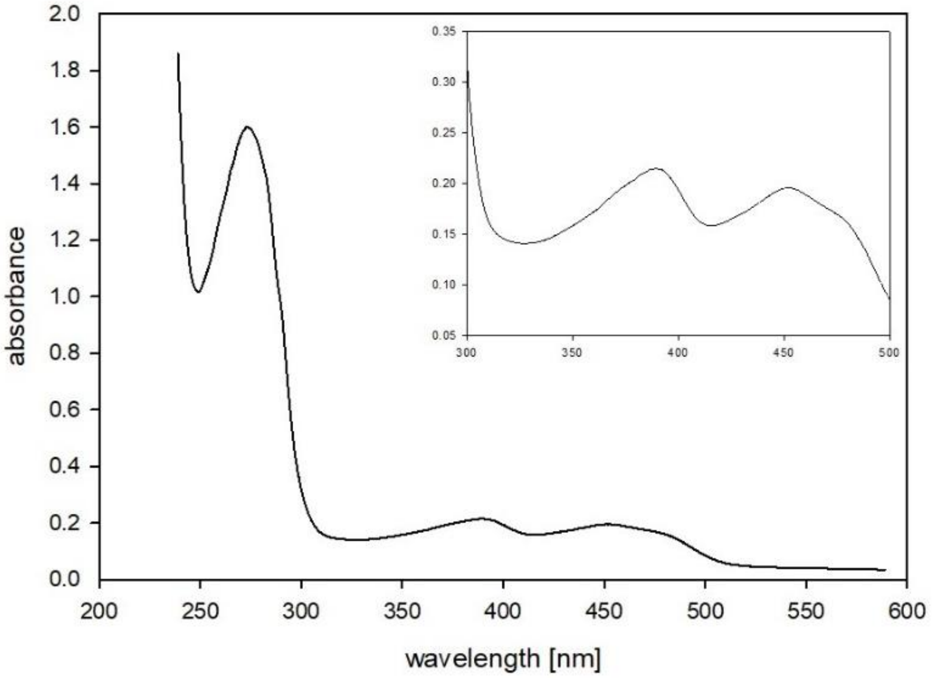

Figure S3. UV-Vis spectrum of a purified *M*POx sample.

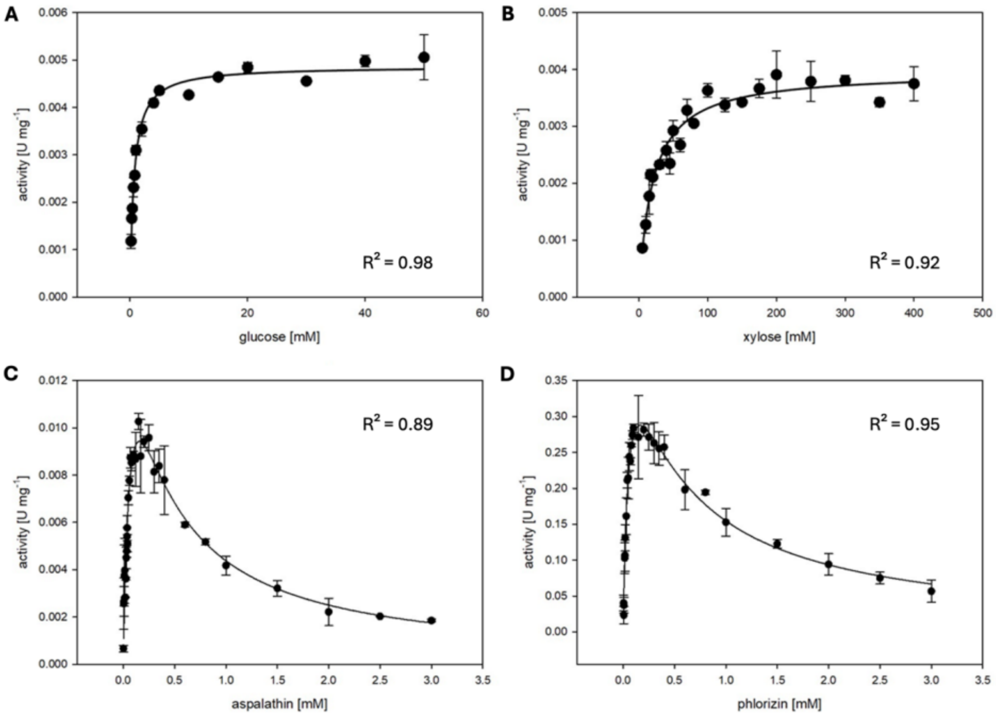

Figure S4. Michaelis-Menten curves for different substrates of *M*POx. Non-linear regression curves for determining the apparent steady-state kinetic constants are shown using oxygen as electron acceptor at a fixed concentration (air saturation) at 30°C and pH 7.5 (50 mM Tris-HCl). All data points were measured in triplicates. (**A**) D-glucose, (**B**) D-xylose, (**C**) aspalathin, (**D**) phlorizin.

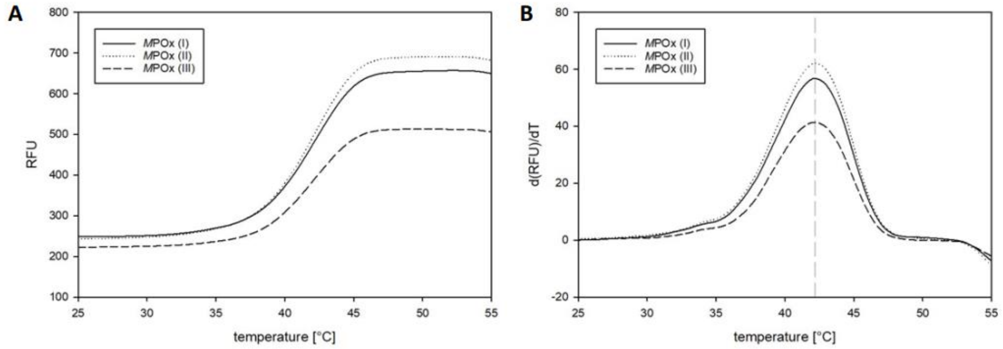

Figure S5. Determination of the melting temperature, measured in triplicates. The curves show (**A**) the relative fluorescence units versus temperature, (**B**) the first derivative. The dashed line indicates the peak maximum at 42.2 °C, which equals the melting temperature.

```
MPOx     MT-----------------------------------------DSPL-IAVVGSGPIGSAYARTILEQSPTARVIMLEAG 38
PcPOx    MF---------LDTTPFR------------------------ADEPYDVFIAGSGPIGATFAKLCVD--ANLRVCMVEIG 45
TmPOx    MSTSSSDPFFNFAKSSFRSAAAQKASASSLPPLPGPDKKVPGMDIKYDVVIVGSGPIGCTYARELVG--AGYKVAMFDIG 78
MtCarA   MSTRV-------------------------------------YPAQVDVAIVGSGPAGATYARILSERASSATIAMFEVG 43
PsPOx    MSGHR-------------------------------------YPAAVDVAIVGSGPTASAYARILSEEAPGATIAMFEVG 43
MPOx2    MT-RS-------------------------------------YPAEVDVVIVGSGPAGATYARVLSERAPHARIALFEVG 42

MPOx     PQVT--------TP---------------AGSSVRNIVDPDEK-ARAREMSQGPQAGAFRESLGIPAGAVVEGMFTARQG 94
PcPOx    AADSFTSKPMKGDPNAPRSVQFGPGQVPIPGYHKKNEIEYQKDIDRFVNVIKGALSTCSIPTSNNHIATLDPSVVSNSLD 125
TmPOx    EIDSGLK----------------------IGAHKKNTVEYQKNIDKFVNVIQGQLMSVSVPVNTLVVDTLSPT--SWQAS 134
MtCarA   PTVS--------DP---------------PGAHVKNIADADER-AHAQRRSEGPHA---REDDDRVGGIVKSAQRRARPG 96
PsPOx    PTVS--------NP---------------PGAHVKNIEDPDSR-SLAQRASEGPGA---GAATVNSPGAVKSGERRARPG 96
MPOx2    PTVT--------DP---------------PGAHVKNIADPSLR-AEAQRRSEGPRGG--TAATVASPGAVKSGERRGRPG 96

MPOx     THLLDFGGEGSAHA-ATFPAAAAATNVGGQGAHWTCAIPRP--------AFSEKLPFIDD-AE-----WEDLIATAEGLL 159
PcPOx    KPFISLGKNPAQNPFVNLGAEAVTRGVGGMSTHWTCATPEFFAPADFNAPHRERPKLSTDAAE-DARIWKDLYAQAKEII 204
TmPOx    TFFVRNGSNPEQDPLRNLSGQAVTRVVGGMSTHWTCATPRF--------DREQRPLLVKDDADADDAEWDRLYTKAESYF 206
MtCarA   TYLLESGYQADGED--GLPVAAFSSNVGGMAAHWTGACPRP--------NDSERIGFLDETGE-----LDELLSEGERLL 161
PsPOx    TYLLQDGYAFPGED--GMPVAAMSSNVGGMAAHWTAACPRP--------GGKERIPFLPD--------LEELLNDADRLL 158
MPOx2    TFLLEEGFAADGED--GLPVLAMSSNVGGMSAHWTGACPRP--------NESERIGFIDD--------LDELLAEADRLL 158

MPOx     HQQSAAFSASPIGAAIRSLLEGEFGAELPDGYGVSTLPVAGDPQPDGSML-WGGADTVLG--PLLTEGGDLASRFELRDL 236
PcPOx    GTSTTEFDHSIRHNLVLRKYNDIFQKEN-VIREFSPLPLACHRLTDPDYVEWHATDRILE--ELFTDPVK-RGRFTLLTN 280
TmPOx    QTGTDQFKESIRHNLVLNKLTEEYKGQ----RDFQQIPLAATRRS-PTFVEWSSANTVFDLQNRPNTDAP-EERFNLFPA 280
MtCarA   GVTTDAFDASPYAGIVRERLAAVEDAHRDADERVQRMPLAVHRRDDGPLV-WSGSDVVLG------DITRGNPNFTLFDE 234
PsPOx    GVTTHAFDGAPFSDLVRERLAAVVDQGRTPAFRVQPMPLAVHRRQDGALV-WSGSDVVMG------EATRDNPQFELFDE 231
MPOx2    GVTTDAFEGAPFSDLVRERLATTADEGREPHARVQRMPLAVHRRDDGALV-WSGADVVFG------DETRANPHFTLFDD 231

MPOx     TLVRRIE------HEDGMVSGVTIQDLR--------TGEQSVLFADAVVIAADAFRSPQLLWASGI----RPAP------ 292
PcPOx    HRCTKLVFKHYRPGEENEVDYALVEDLLPHMQNPGNPASVKKIYARSYVVACGAVATAQVLANSHI----PPDDVVIPFP 356
TmPOx    VACERVV----RNALNSEIESLHIHDLI--------SGDRFEIKADVYVLTAGAVHNTQLLVNSGFGQLGRPNP------ 342
MtCarA   SLVTRVL------VEDGRAAGVVVTDVR--------TGERRDVRARFVVVAADALRTPQLLWASGI----RPDA------ 290
PsPOx    SLVTRVL------VEDGTAAGVEVQDRR--------SGDTYQVAARYVVVGADALRTPQLLWASGI----RPDA------ 287
MPOx2    SLVTAVL------VEDGHAAGVRVNDRR--------SGDEHEVRARAVVIAADALRTPQILWASGI----RPDA------ 287

MPOx     ----------------------LGRYLTEHHVVITTIALDAPRMADLVTEE--ELEGELARRAMNPADPVAAV------N 342
PcPOx    GGEKGSGGGERDATIPTPLMPMLGKYITEQPMTFCQVVL-DSSLME-------VVRNPP----------WPGL------D 412
TmPOx    -------------ANPPELLPSLGSYITEQSLVFCQTVM-STELIDSVKSDM-TIRGTPGE-LTYSVTYTPGASTNKHPD 406
MtCarA   ----------------------LGRYLNDQAQIVF-----AVRMRDFTP-----VVDADGV-PQTGLSEYTGV------T 331
PsPOx    ----------------------LGRYLNDQAQVVF-----ASRLRDVQP------EDAPAA-ANGALSEQSGV------A 327
MPOx2    ----------------------LGRYLNDQTQVVY-----ATRIRDIAAAGMGAAGGADAP-PAAGIGATSGV------T 333

MPOx     R------------------IPFSEP----------DHPFSLQVMYAENPPFQL----DPAHPAAGNRWGYVNMGYGVRKH 390
PcPOx    WWKEKVARHVEAFPNDPIPIPFRDPEPQVTIKFTEEHPWHVQIHRDAFSYGAVAENMDTRV--------IVDYRFFGYTE 484
TmPOx    WWNEKVKNHMMQHQEDPLPIPFEDPEPQVTTLFQPSHPWHTQIHRDAFSYGAVQQSIDSRL--------IVDWRFFGRTE 478
MtCarA   W------------------VPFTD-----------DMPFHGQVMQLDASPVKLADD-DPAAPGS-----IVGLGLFCAKD 376
PsPOx    W------------------VPYTD-----------EAPFHGQIMQLDASPVPLADD-DPIVPGS-----IVGLGLFCAKD 372
MPOx2    W------------------VPYTD-----------AKPFHGQVMQLDASPIALAED-DPAVPGS-----IVGLGLFCAKD 378

MPOx     PRIEDGVSFSDDELDYRGLPNMTIEYALTEVEEAE-IAEATERLRRAGRALGAFV--AEPRLLPNGSSLHYMGTVRCG-T 466
PcPOx    PQEANELVFQQHYRDAYDMPQPTFKFTMSQDDRAR-ARRMMDDMCNIALKIGGYLPGSEPQFMTPGLALHLAGTTRCG-L 562
TmPOx    PKEENKLWFSDKITDAYNMPQPTFDFRFPAGRTSKEAEDMMTDMCVMSAKIGGFLPGSLPQFMEPGLVLHLGGTHRMG-F 557
MtCarA   LQASDRVAFSDSDVDGYGMPAMQLHYTLSDRDHAT-IDRAKAEIVRLGKAIGDPL-DDRPFVMPLGASLHYQGTVRMGLA 454
PsPOx    LQREDRVAFDDDTRDSYGLPAMRIHYRLTERDHVV-LDRARQEIVRLGKAVGEPL-DERPFVLPPGASLHYQGTTRMGET 450
MPOx2    LQREDRVDFSDERLDGYGLPAPQIHYRLTARDHEV-LDRAREEIVRLAAAVGEPL-GERPFTLPFGASLHYQGTTRMGAV 456

MPOx     DPAV--SVADPYSRVWGFENLVVGGNGLIPTANTMNPTLMSVAIAVRGARALAAHVGSRT--------------AD 526
PcPOx    DTQK--TVGNTHCKVHNFNNLYVGGNGVIETGFAANPTLTSICYAIRASNDIIAKFGR----------------HR 620
TmPOx    DEKEDNCCVNTDSRVFGFKNLFLGGCGNIPTAYGANPTLTAMSLAIKSCEYIKQNFTPSPFTSE----------AQ 623
MtCarA   DDGA--SVCSPDSEVWGAPGLFVAGNGVIPTATACNPTLTGVALAVRGARHIADEITADLASVKLAAALEHHHHHH 528
PsPOx    DDGE--SVCSPDSQVWQVPGLFVAGNGVIPTATACNPTLTSVALAVRGARKIAEEITSSLLMSESDNRL-----SK 519
MPOx2    DDGE--SVCGPNSEVWGVSGLYVAGNGVIPTSTACNPTLTSVALAVRGARALAAHL-------------------- 510
```

Figure S6. Multiple sequence alignment. The box marks the amino acids in alignment with the substrate loop of *Ps*G3Ox [16]. Identical amino acids have a dark grey background while similar amino acids are shaded in light grey.

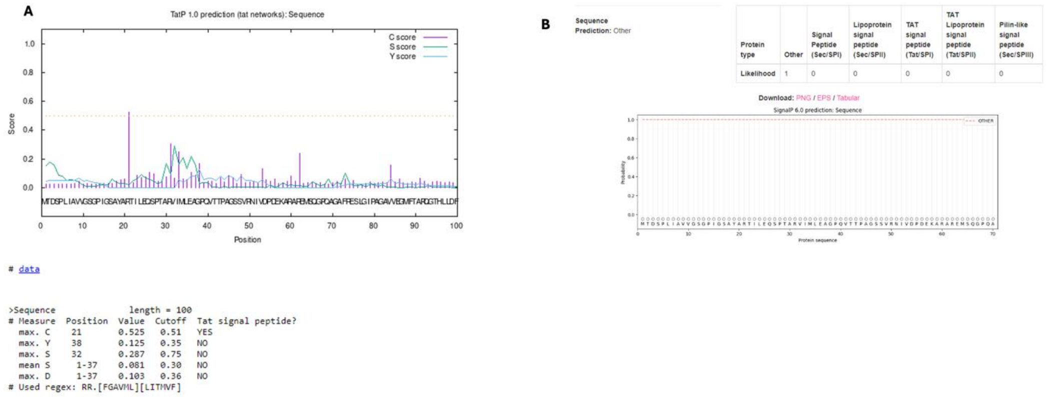

Figure S7. Signal peptide predictions. Predications were made with (**A**) TatP and (**B**) SignalP.

Table S1. Sequence identities of *M*POx and *M*POx2 with other members of the actinobacterial sequence space (*Ka*POx, *Sc*POx, *Ps*G3Ox, *Mt*CarA).

	***M*POx**	***M*POx2**
*M*POx	-	40.27%
*Sc*POx	34.51%	34.99%
*Ka*POx	35.56%	35.92%
*Mt*CarA	40.19%	69.92%
*Ps*POx	40.34%	73.23%
*M*POx2	40.19%	-
